# Hypertrophic osteoarthropathy as the cause of a super scan of the bone in a patient with prostate cancer: a case report

**DOI:** 10.1186/1752-1947-2-104

**Published:** 2008-04-07

**Authors:** Boris LJ Kanen, Ruud JLF Loffeld

**Affiliations:** 1 Department of Internal Medicine, Zaans Medical Center, Zaandam, The Netherlands; 2 Department of Endocrinology, VU University Medical Center, Amsterdam, The Netherlands

## Abstract

**Introduction:**

Prostate cancer is known to have a tendency to metastasize to bone. Skeletal scintigraphy can be used to show multiple lesions. Diffuse metastasis, which is not infrequent in prostate cancer, can also be suspected on the basis of a 'super scan'. However, this phenomenon in nuclear medicine has several other causes that need to be considered.

**Case presentation:**

A patient with a history of prostate cancer presented with pleural fluid, peripheral edema and bone pain. A super scan of the bone was found which suggested diffuse skeletal metastasis of the prostate cancer but the patient also had a prostate specific antigen level which was not compatible with this diagnosis. Further investigations revealed the paraneoplastic phenomenon of hypertrophic osteoarthropathy, related to an incurable carcinoma of the lung, to be the cause of the super scan.

**Conclusion:**

A super scan is characterized by a high bone to soft tissue ratio on skeletal scintigraphy, with a uniform symmetrical increase in bone uptake and diminished to absent renal visualization ('absent kidney sign'). It can be seen in a variety of diseases in which there is a diffusely increased bone turnover. Diffuse skeletal metastasis of a well-differentiated prostate carcinoma is unlikely to be the cause of a super scan when the prostate specific antigen level is not elevated. This is the first report of a super scan due to pulmonary hypertrophic osteoarthropathy which can be seen in lung carcinoma and other pulmonary diseases.

## Introduction

Prostate cancer has a high tendency of metastasizing to the skeleton. In fact, many cases of this cancer are diagnosed because of the detection of bone metastasis from a primary tumor of unknown origin at the time of presentation. The majority of patients with metastatic prostate cancer will have multiple skeletal lesions. However, diffuse metastases are also described. These patients have a so-called super scan of the bone. Presence of a super scan is not pathognomic for diffuse bone metastasis. The differential diagnosis is wider as is described in this case report.

## Case presentation

A 81-year-old man with an adenocarcinoma of the prostate diagnosed one year earlier presented with a five month history of gradually progressive complaints of dyspnea. At the time of diagnosis of the prostate cancer, there had been no signs of metastases and since it was an asymptomatic grade 2 prostate cancer in a man of advanced age, a watchful waiting policy was followed. The medical history revealed hypertension and a transurethral resection of the prostate six years before presentation. The patient complained of dyspnea, progressive peripheral edema, orthopnea, and painful knees and thighs which made walking extremely difficult. There had been weight loss of ten kilograms over six months, with associated loss of appetite. No thoracic pain, hemoptysis or other pulmonary or cardiac complaints were present. The patient had been a heavy smoker for fifty years.

On admission his blood pressure was 150/80 mmHg with an irregular pulse of 96 per minute, temperature 36.2°C, and he had a normal central venous pressure. The heart sounds were normal. Percussion and auscultation of the left lower lung revealed dullness with diminished breath sounds. These signs were indicative of pleural effusion. The liver was not enlarged. There was pitting edema especially at the lower extremities, but also of both hands, which were also noted to be remarkably large. Percussion of, and axial pressure on, the vertebrae was not painful. The patient refused rectal examination because of painful earlier experiences.

Laboratory examination revealed the following data: ESR 35 mm in the first hour (normal: <7), CRP 134 mg/l (normal <10), hemoglobin 6.3 mmol/l (normal: 8.9–10.7) with a MCV of 82 fl (normal: 80–100), leukocytes 8.6 × 10^9^/l (normal: 4.5–10.0) with 90% neutrophilic granulocytes (normal 40–70), normal blood platelets, electrolytes and liver enzymes. Creatinine was 77 μmol/l (normal: 64–108), alkaline phosphatase was elevated at 285 U/l (normal: 40–120), calcium was 1.95 mmol/l (normal: 2.15–2.68) with an albumin of 23.9 g/l (normal: 35–50 g/l) and a normal phosphate. Blood gas analysis showed a chronic compensated respiratory acidosis with an oxygen saturation of 80%.

Electrocardiography showed atrial fibrillation with a left bundle branch block, similar to earlier ECGs. Chest X-ray revealed a large amount of pleural fluid on the left side and an enlarged heart without signs of vascular redistribution. There were no signs of tumor or pulmonary metastasis on chest X-ray.

Analysis of the pleural fluid was performed. A total amount of 4.5 liters was evacuated. Cytological and biochemical analysis showed only lymphocytosis with no signs of malignancy or bacterial infection. Auramin and Löwenstein cultures were negative. An echocardiography showed good left ventricular function. Ultrasound investigation of the abdomen showed a dilated inferior caval vein without other abnormalities. The entire presentation was compatible with right-sided heart failure in a patient with probable pulmonary hypertension. On Computed Tomography Angiography (CTA) there were no pulmonary embolisms visible but a large amount of pleural fluid was seen in the left pleural cavity.

**Figure 1:**
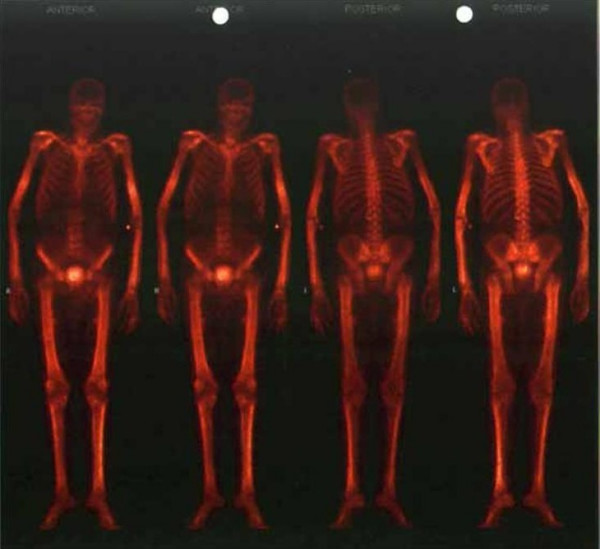
Skeletal scintigraphy showing diffusely increased uptake and the absent kidney sign (a super scan).

Because of the elevated alkaline phosphatase, the bone pains and the previously diagnosed prostate cancer, skeletal scintigraphy was performed. It showed a 'super scan', meaning there was diffuse uptake throughout the entire skeleton. This was judged as fitting diffuse skeletal metastasis of the prostate cancer [Figure 1]. However the prostate specific antigen was within the normal range at 1.4 μg/l (normal < 4.4)

**Figure 2:**
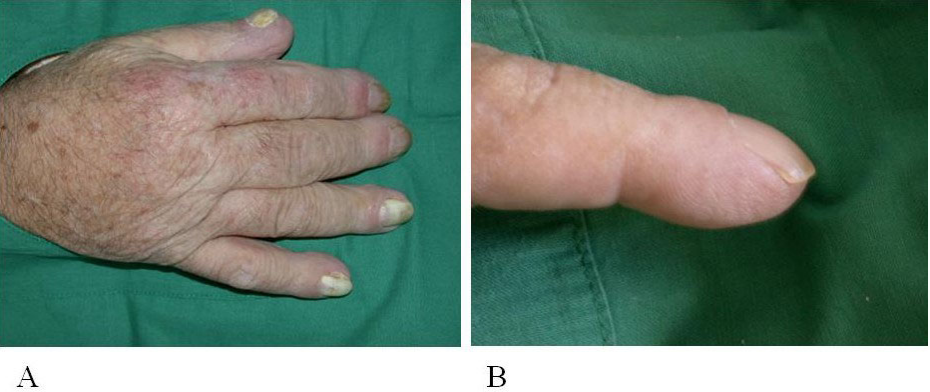
**A. Large sized right hand with edematous swelling.** B. Periungual erythema and clubbing.

**Figure 3:**
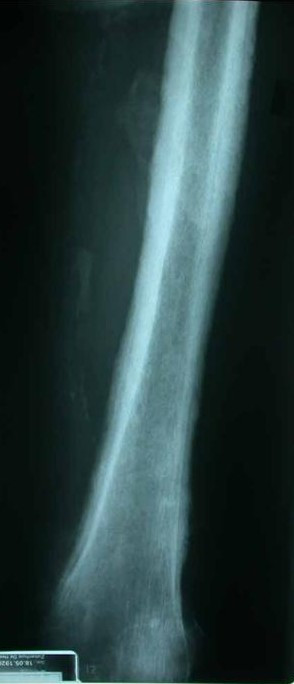
X-ray of the femur showing extensive characteristic periosteal bone apposition.

With the remarkably large hands in mind, additional investigations were carried out [Figure 2]. A bone marrow examination showed no marrow disease nor malignancy. X-ray of the hands, humeri, femora and pelvis revealed extensive subperiosteal bone appositions compatible with generalized hypertrophic osteoarthropathy [Figure 3a/b]. Repeat of the earlier performed CTA indeed now showed a fluid-containing cavity in the lower left lobe surrounded by a large amount of pleural fluid at that side suggestive of a lung cancer. Bronchoscopy confirmed this diagnosis. The left main bronchus was stenotic with tumor totally occluding the left lower lobe and almost occluding the left upper lobe. Histological examination was not possible due to technical difficulties during the procedure. The diagnosis of incurable bronchial carcinoma with hypertrophic osteoarthropathy was made with the prostate cancer as an "innocent" bystander. Since the patient was rapidly deteriorating palliative care was given. The patient died several weeks after admission. Post mortum examination confirmed the clinical diagnosis. There was a large undifferentiated non-small cell lung carcinoma with a diameter of 10 cm and extension in the adventitia of the esophagus and lymphatic metastasis in the hili and mediastinum. Three liters of tumor-positive pleural fluid and extensive hypertrophic osteoarthropathy was seen without distant metastasis.

## Discussion

A super scan is characterized by a strikingly high bone to soft tissue ratio on skeletal scintigraphy, with a uniform symmetrical increase in bone uptake and diminished to absent renal visualization ('absent kidney sign'). It can be seen in a variety of diseases in which there is diffusely increased bone turn over. Diffuse skeletal metastasis, as can be observed from primary tumors of the breast, lung, prostate, bladder and lymph nodes, is the most frequent cause. Other causes are secondary hyperparathyroidism, Paget disease, myelofibrosis and metabolic bone disease.

Technetium-99m-labeled methylene diphosphonate (^99m^Tc-MPD) bone scintigraphy performed in patients presenting with prostate cancer shows metastases in 10–50%. It has a false negative rate of 1–5%, mostly being due to a super scan [1]. When caused by prostate cancer, super scans are found exclusively in histologically high-grade forms.

Hypertrophic Osteoarthropathy (HOA), also known as the classical Pierre Marie-Bamberger syndrome, is a systemic disorder of the bones, joints and soft tissues that develops in association with other disease processes. It is characterized by several or all of the following signs [2]: clubbing of the digits, periosteal new bone formation, particularly involving the long bones of the distal extremities, symmetric arthritis-like changes in the joints and periarticular tissues (ankles, knees, wrists, and elbows), increased thickness of the subcutaneous soft tissues in the distal one-third of the arms and legs and sometimes of the facial tissues, which may simulate acromegaly [3] and finally, neurovascular changes of the hands and feet including chronic erythema, paresthesia and increased sweating.

Most commonly it is associated with an intrathoracic malignancy, which can be carcinoma of the lung as well as pulmonary metastasis of other tumors and Hodgkin's disease involving the mediastinum. HOA is also frequently seen in severe cystic fibrosis, bronchiectasis, chronic empyema and lung abscess and occasionally in certain liver disorders' [4]. In some instances it may present without any underlying illness when it is called primary, idiopathic or the hereditary form of HOA in which bone and joint pain tends to be less, and the furrowing of the face and scalp tends to be more severe. Diagnosis must be based on global assessment of the clinical, laboratory and radiographic findings rather than the presence of one abnormality, since there are well-documented cases that lacked radiographically detectable periostitis [5]. Blood studies are usually unaffected by HOA except that often an elevated ESR of more than 50 mm/h is seen and in advanced cases an elevated alkaline phosphatase level can be found [6]. The incidence of clinically apparent HOA in patients diagnosed with lung cancer is approximately 4–5% [7]. The etiology is still poorly understood. Several pathogenetic theories have focused on the vascular changes and proliferation that might be caused by circulating growth factors that normally are inactivated in the lungs. Pulmonary shunting caused by the several disease processes that are associated with HOA causes a faulty pulmonary clearance of macrothrombocytes, which release growth factors in the systemic circulation. Elevated levels of platelet-derived growth factor (PDGF), endothelin-1 (ET-1), β-thromboglobulin (β-TG) and vascular endothelial growth factor (VEGF) [8] have all been shown to be elevated in patients with HOA.

HOA has no prognostic significance and early detection may lead to detection of potentially resectable lung carcinoma. Subclinical cases can be diagnosed by radiographs or, with more sensitivity, by skeletal scintigraphy with an incidence in bronchogenic carcinoma of up to 20% [6]. Usually scintigraphic abnormalities are found in the peripheral skeleton and are not easily mistaken for diffuse skeletal metastasis. Its appearance can range from increased 'bracelet-like' appearance to more diffusely increased uptake at the distal ends of the long bones. Although usually located in the peripheral skeleton, it can also affect the skull, claviculae, ribs and scapulae [9,10]. Sometimes along the cortical margins, a 'parallel track sign' due to the periosteal bone formation can be seen. To the best of our knowledge this is the first report of a super scan as the presenting feature of HOA.

This case report clearly illustrates the pitfalls of diagnostic tests. The most important is the interpretation of the bone scan. Although the clinical presentation almost entirely fitted the suggested diagnosis of diffusely metastasized prostate carcinoma as an explanation for the symptoms, signs and bone scintigraphy, it was the normal PSA, which indicated that an alternative diagnosis should be sought. Studies report that a PSA of less than 20 ng/ml has a negative predictive value of 92–95% for the absence of skeletal metastases in patients with well-differentiated (grade 1 and 2) or clinically localized (stages T1–2) prostate cancer. In patients with poorly differentiated (grade 3) or clinically advanced (stages T3–4) tumors it has a negative predictive value of only 70 and 50%, respectively [10,11]. And although advanced technologies are at our disposal, in almost 4.5 liters of pleural fluid no malignant cells were found. Imaging technologies are so refined that peripheral embolism on a CTA can be detected, but gross abnormalities like pleural fluid and atelectasis can cover up a malignant tumor of 10 cm in diameter.

## Conclusion

This case illustrates that a super scan of the bone is a distinct entity in nuclear medicine that can point towards several different bone disorders. Malignancy is a frequent cause, but the most obvious cause in that setting, namely diffuse skeletal metastasis, is not the only one that should be considered. In prostate carcinoma, especially when well-differentiated with a low PSA level, skeletal metastasis is unlikely. As in this case, non-metastatic paraneoplastic, or even benign diagnoses should be considered. To our knowledge this is the first report of a super scan due to extensive HOA. Usually scintigraphic abnormalities are confined to the peripheral skeleton.

In modern day medical practice, a clinician is often confronted with the results of several diagnostic modalities. It remains the task of the clinician to be critical and to interpret the different aspects and findings in relation to each other.

## Competing interests

The author(s) declare that they have no competing interests.

## Authors' contributions

BK and RL have both been involved in the management of the patient as well as writing the case report. Both authors have read and approved the manuscript.

## Consent

Informed and verbal consent was obtained from the patient for both publication and use of the clinical photographs. Written consent was not obtained before death. The patient had no relatives or partner who could be asked to provide written consent.
